# Supplementary material for: Cardiovascular disease risk perception among community-dwelling adults in southwest Nigeria: A mixed-method study
Source: PLoS One. 2024 Nov 12;19(11):e0313578. doi: 10.1371/journal.pone.0313578 (PMC11556699; doi:10.1371/journal.pone.0313578)

**Supplementary File 1**

Supplementary File S1a: Knowledge about cardiovascular disease

**
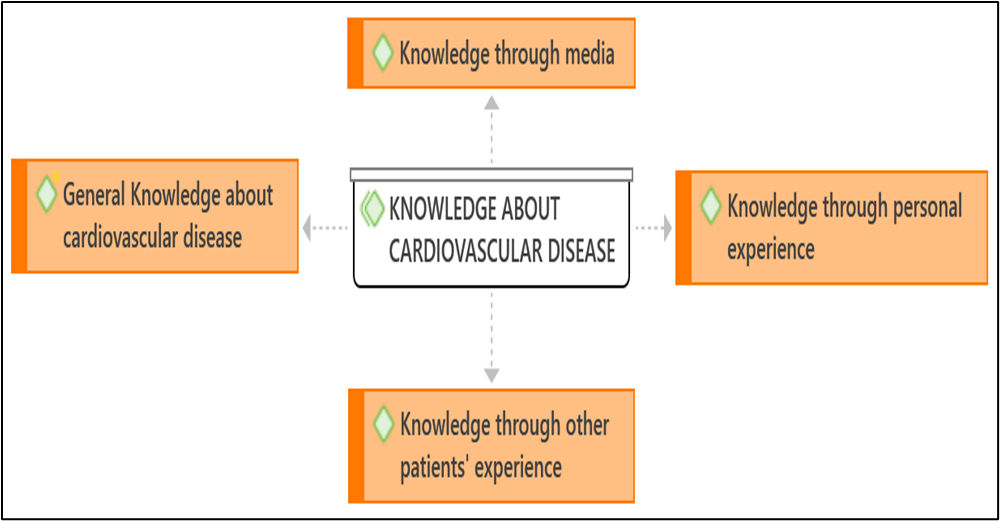
**

Supplementary File S1b: cardiovascular disease risk factors


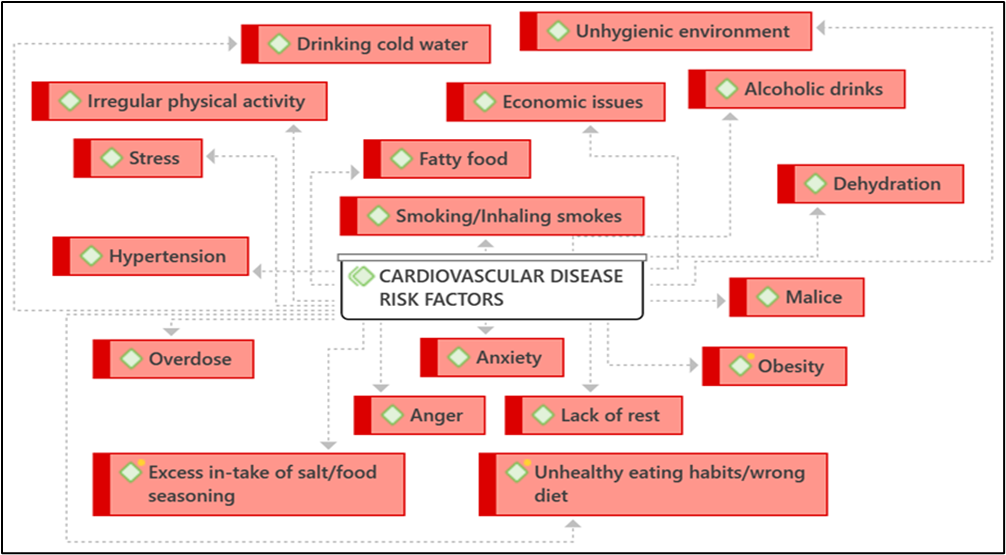


Supplementary File S1c: Dangers of having cardiovascular disease


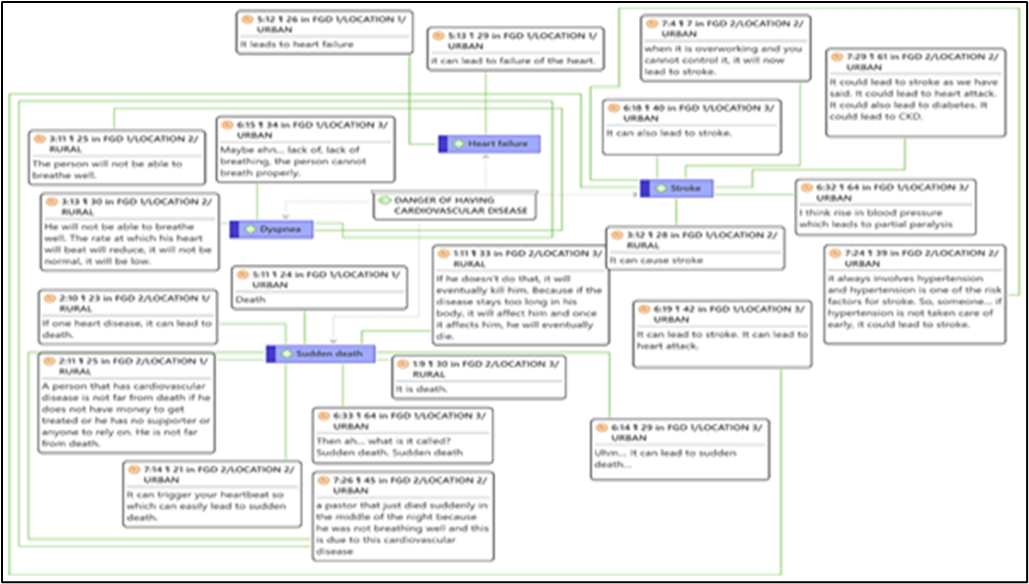


Supplementary File S1d: Perception of vulnerability to cardiovascular


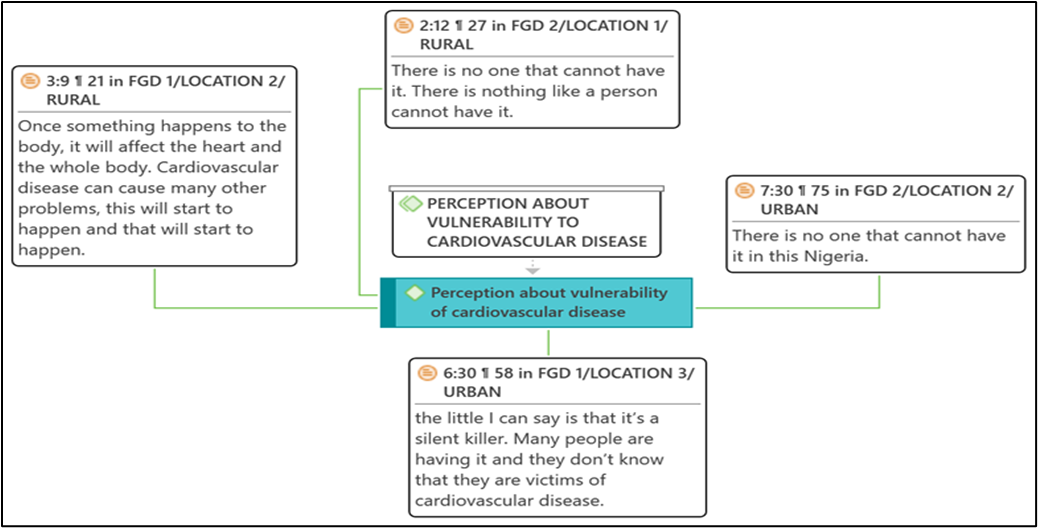


Supplementary File S1e: Prevention of cardiovascular disease


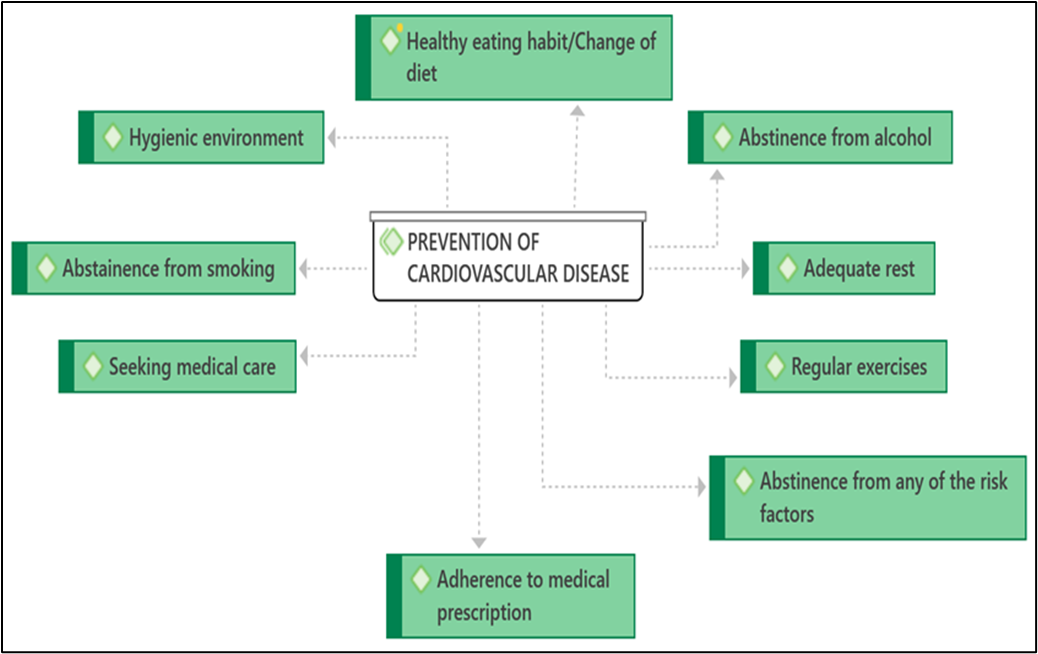

Supplement: S1 File — S1a –S1e: Knowledge about cardiovascular disease (S1a), Cardiovascular disease risk factors (S1b), Dangers of having cardiovascular disease (S1c), Perception of vulnerability to cardiovascular (S1d), and Prevention of cardiovascular disease (S1e). (DOCX) [file pone.0313578.s001.docx]
