# Supplementary material for: Cardiovascular disease risk perception among community-dwelling adults in southwest Nigeria: A mixed-method study
Source: PLoS One. 2024 Nov 12;19(11):e0313578. doi: 10.1371/journal.pone.0313578 (PMC11556699; doi:10.1371/journal.pone.0313578)
Supplement: Attachment — Submitted filename: Paper Review.docx [file pone.0313578.s003.docx]

Paper Review

Thank you for giving me the opportunity to be a part of the publication journey of this article.

Abstract

This is good

BACKGROUND

Line 62. Replace “Theory” with “Model”

Line 69. While it is understandable that the HBM is under the general concept of “Framework”, it is still better to address it by its name “Health Belief Model”

Line 70. Rephrase as “Should first be”

METHODS

Study design: I do not think the ethical approval is expected to be in the study design sub-section. Usually, I know it comes as a sub-section of its own after the data collection and/or analysis sub-section (depending on whether or not you combine it)

Study setting: Niger has six southwest states. Your sentence sounds confusing when you say “Nigeria’s three southwest states”. Rather, say “three of Nigeria’s six southwest states”.

Also in the next sentence, add “in each state” between *Communities* and *Were*

Under the study setting, I recommend you include a national map calling out each of the selected states with a unique colour

Study Participants: Add “and Selection” after *Participants.*

Justify how you determined the participants had no history of CVD. Did you ask or were they tested?

It is not clear how you have employed multi-stage sampling. I think it will be good if you break it down properly. Your study is in the southwest, did you purposively select the three states, or balloting as was presented? How did you select the communities without calling out the local government areas involved, or are the communities the LGA? If the names listed in the study settings are the LGA, then you will still include communities you visited to assess your population.

Sample size estimation: I recommend you add the number of FGDs with 8 participants and those with 10 participants. Alternatively, you can add this under the Instrument and Procedure sub-section. It will also be good you add why and in which of the states this had occurred.

Instrument and Procedure: It is not clear whether the FGD was moderated in English and/or Yoruba, particularly because you mentioned that rural areas were involved. Members of the rural community are probably unfamiliar with the term cardiovascular disease. This also means that in the data analysis section, there is a need to state whether transcripts were translated.

RESULTS

Socio-demographics: I recommend you either complete the information for the qualitative participants or remove it from the table. The editor can advise better.

Qualitative Results: I recommend you use 1.0 line spacing for your excerpts. That spacing makes them easily distinctive from your writing.

Cardiovascular diseases factor: Under the first sub-theme, you were able to do a brief introduction for diet-related factors sub-sub-themes, but not the other two. I think it will be helpful if you do that.

Sub-theme 2.1 was not boldened but sub-theme 2.2 was. That is a bot confusing. Kindly maintain consistency.

Line 362. Rephrase the second sentence to read well

DISCUSSION

Before introducing your study findings, it will be good you restate the overall aim of the study once again, as an introductory sentence(s) to the discussion section. It is evident that the first two sentences currently speak to your research questions 1 and 2.

Generally, much of the discussion section is premised on the quantitative findings, and a limited part of the qualitative findings were discussed.

Additionally, a sub-heading should be dedicated to the study limitation and not combined with the discussion.

RECOMMENDATIONS

A sub-section should be dedicated to this as well. It should be removed from the conclusion.

CONCLUSION

This reads good

REFERENCING

This is also good
