# Supplementary material for: Cardiovascular disease risk perception among community-dwelling adults in southwest Nigeria: A mixed-method study
Source: PLoS One. 2024 Nov 12;19(11):e0313578. doi: 10.1371/journal.pone.0313578 (PMC11556699; doi:10.1371/journal.pone.0313578)
Supplement: Attachment — Submitted filename: Response to Reviewers_270924.docx [file pone.0313578.s004.docx]

**Response to Reviewers**

Paper Review

Thank you for giving me the opportunity to be a part of the publication journey of this article.

Abstract

This is good

**Response**: Thank you.

BACKGROUND

Line 62. Replace “Theory” with “Model”

**Response:** Done. Please see introduction section, line 81, page 4.

Line 69. While it is understandable that the HBM is under the general concept of “Framework”, it is still better to address it by its name “Health Belief Model”

**Response**: Done. Please see introduction section, line 99, page 5.

Line 70. Rephrase as “Should first be”

**Response:** Done. Please see introduction section, line 100, page 5.

METHODS

Study design: I do not think the ethical approval is expected to be in the study design sub-section. Usually, I know it comes as a sub-section of its own after the data collection and/or analysis sub-section (depending on whether or not you combine it)

**Response:** We have moved ethical approval below data analysis section. Please see ethical approval section, lines 218-2205, pages10-11.

Study setting: Niger has six southwest states. Your sentence sounds confusing when you say “Nigeria’s three southwest states”. Rather, say “three of Nigeria’s six southwest states”.

Also in the next sentence, add “in each state” between *Communities* and *Were*

**Response:** Done. Please see the methods section, lines 128-129, page 6.

Under the study setting, I recommend you include a national map calling out each of the selected states with a unique colour

**Response:** We opined that this may not be necessary. The mention of the selected states is sufficient.

Study Participants: Add “and Selection” after *Participants.*

**Response:** Done. Please see the methods section, line 135, page 7.

Justify how you determined the participants had no history of CVD. Did you ask or were they tested?

**Response:** Participants were asked if they were told to have or had received treatment for CVD to screen them for the presence of CVD. Please see the methods section, lines 138-139, page 7.

It is not clear how you have employed multi-stage sampling. I think it will be good if you break it down properly. Your study is in the southwest, did you purposively select the three states, or balloting as was presented? How did you select the communities without calling out the local government areas involved, or are the communities the LGA? If the names listed in the study settings are the LGA, then you will still include communities you visited to assess your population.

**Response:** We have provided additional information about multi-stage sampling. Please see the methods section, lines 140-145, page 7.

Sample size estimation: I recommend you add the number of FGDs with 8 participants and those with 10 participants. Alternatively, you can add this under the Instrument and Procedure sub-section. It will also be good you add why and in which of the states this had occurred.

**Response:** We opined this may not be necessary. The information looks like results which have been provided in results section. Please see the results section, lines 231-233, page 11.

Instrument and Procedure: It is not clear whether the FGD was moderated in English and/or Yoruba, particularly because you mentioned that rural areas were involved. Members of the rural community are probably unfamiliar with the term cardiovascular disease. This also means that in the data analysis section, there is a need to state whether transcripts were translated.

**Response:** The FGD was moderated in English and/or Yoruba as applicable in each location. The transcripts were translated to English before analysis. Please see data analysis and methods sections, lines 181 & 206, pages 9-10

RESULTS

Socio-demographics: I recommend you either complete the information for the qualitative participants or remove it from the table. The editor can advise better.

**Response:** We have removed the information for the qualitative participants from Table 1 and reported it in words. Please see the results section, lines 231-233, page 11.

Qualitative Results: I recommend you use 1.0 line spacing for your excerpts. That spacing makes them easily distinctive from your writing.

**Response:** Done. Please see the results section, lines 296-508, pages 18-24.

Cardiovascular diseases factor: Under the first sub-theme, you were able to do a brief introduction for diet-related factors sub-sub-themes, but not the other two. I think it will be helpful if you do that.

**Response:** Done as suggested. Please see the results section, lines 351-388, pages 19-20.

Sub-theme 2.1 was not boldened but sub-theme 2.2 was. That is a bot confusing. Kindly maintain consistency.

**Response:** We have unboldened sub-theme 2.2. Please see the results section, lines 394, page 21

Line 362. Rephrase the second sentence to read well

DISCUSSION

Before introducing your study findings, it will be good you restate the overall aim of the study once again, as an introductory sentence(s) to the discussion section. It is evident that the first two sentences currently speak to your research questions 1 and 2.

**Response:** We have added a statement about overall aim as suggested. Please see discussion section, lines 511-512, page 24.

Generally, much of the discussion section is premised on the quantitative findings, and a limited part of the qualitative findings were discussed.

**Response:** We have made attempt to improve the discussion. Please see the discussion section, lines 511-601, pages 24-28.

Additionally, a sub-heading should be dedicated to the study limitation and not combined with the discussion.

**Response:** Done. Please see the limitation section, line 603, page 28.

RECOMMENDATIONS

A sub-section should be dedicated to this as well. It should be removed from the conclusion.

**Response:** Done. Please see recommendation section, Line 626, page 29.

CONCLUSION

This reads good

**Response:** Thank you.

REFERENCING

This is also good

**Response:** Thank you.

Reviewer #1: This study offers valuable insights into the perception of cardiovascular disease risks among community-dwelling adults in Southwest Nigeria. The use of a mixed-methods approach enriches the study, providing both quantitative data and qualitative insights that complement each other. The study is well-organized, with each section contributing to a coherent narrative that underscores the importance of addressing CVD risk perception in public health interventions.

However, there are areas where the study could be strengthened. The introduction could better articulate the research questions and the gaps in the literature that the study aims to fill. The methodology section is detailed but could benefit from more transparency about the sampling process and potential biases. The results are clearly presented, but additional statistical details and qualitative data would enhance the robustness of the findings. The discussion and conclusion are well-argued but could provide deeper analysis and more specific recommendations.

In conclusion, this study makes a significant contribution to the understanding of CVD risk perception in Nigeria, with important implications for public health policy and practice. With some refinements, particularly in the areas of methodology, analysis, and recommendations, the study could offer even greater insights and practical guidance for improving cardiovascular health in the region.

**Response:** Thank you for the comments. We have addressed these comments in the appropriate sections of the manuscript. The changes are tracked.

Reviewer #2: Thank you for giving me the opportunity to review this paper. The paper has a nice read, and it was quite enlightening for me in terms of providing knowledge on cardiovascular diseases. it also provides insight into the risk perception of CVD among community dwelling adults. I believe the authors have demonstrated good knowledge of the understand of the study findings. I also appreciate the diagrammatic presentation of the qualitative findings from the thematic breakdown and analysis of the discussion with the participants.

**Response:** Thank you.

**Additional comments from manuscript**

Abstract

This portion could further benefit from providing more context about the prevalence of CVD in South-west Nigeria. This would help emphasize the importance of the study and its potential impact.

**Response:** A statement about the current prevalence of CVD and its burden has been added. Please see the abstract section, line 26, page 2.

Introduction

1. More specific information is required in this section in order to appropriately contextualize CVD burden and its risk factors in Nigeria.

Additionally, the topic allows a more robust discussion on existing interventions or educational campaigns in Nigeria, and how they might have impacted CVD risk perception.

**Response:** We have provided specific information about CVD burden and its risk factors in Nigeria and measures to combat the rising rate and its impact on CVD risk perception. Please see the introduction section, lines 64-80, pages 3-4.

2. Not enough details on how each component of the HBM (perceived susceptibility, perceived severity, perceived benefits, perceived barriers, cues to action, and self-efficacy) relates to the study's focus on CVD risk perception. A brief elaboration on how these constructs are expected to influence CVD risk perception and subsequent behaviors would provide a clearer theoretical framework.

**Response:** We have provided a brief elaboration on how these constructs are expected to influence CVD risk perception and subsequent behaviors. Please see the introduction section, lines 81-94, pages 4-5.

Methods

Setting: This section would benefit from more detail on the multi-stage sampling technique. Specifically, how were the specific communities (e.g., Oluyole, Egbeda) chosen within each state? Was the selection based on specific criteria (e.g., population size, accessibility)? Clarifying this would help assess the representativeness of the sample.

**Response:** We have provided additional information on the multi-stage sampling technique under the study participants section to avoid repetition. Please see the methods section, lines 140-145, page 7

Study participants: The process of participant recruitment could be explained in greater detail. For example, how were individuals approached in public places like worship centers? Were there any challenges in recruiting participants from rural areas versus urban areas? Providing this information would help readers understand potential biases in the sample.

**Response:** Churches and mosques with a higher population and easy accessibility were chosen in each selected community. The religious leaders in each community helped in facilitating contact with prospective participants, and that enabled easy access to capture their data on the day of worship. Please see the methods section, lines 147-150, page 7.

Given that the study involves diverse populations, including rural and urban dwellers, it would be useful to mention any culturally sensitive approaches taken during data collection, particularly in the FGDs. This could include considerations of language use, gender dynamics, or local customs that might influence participation or responses.

**Response:** We only considered how the language of the interview might have impacted on the data collection and thus, English and/or Yoruba were employed. We have added the information. Please see the methods section, line 181, page 9.

Sample size estimation: While a sample size calculation was performed, the rationale for using a prevalence of 54.1% remains unclear.

**Response:** A previous study from southwest Nigeria reported 54.1% poor CVD risk perception. The study was cited (Ref. 13)

A brief discussion on how the power calculation informed the final sample size is required.

Were any adjustments made based on anticipated response rates or potential dropouts? This would enhance the transparency of the study design.

**Response:** The quantitative study was cross-sectional in nature, and we set to recruit at least 402 participants in each state (201 each from rural and urban community). Please see methods section, lines 159-160, page 8.

Instruments and procedure: The cross-cultural adaptation of the Perception of Risk of Heart Disease Scale (PRHDS) into Yoruba is commendable, but the process could be described in more detail. For instance, were there any challenges in adapting certain items culturally or linguistically? How was the validity of the adapted scale assessed? This information will make for a more rounded and robust methods’ section.

**Response:** The detailed description is already provided in another paper under review. To avoid self-plagiarism these details may not be necessary in this paper.

Data analysis:

1. This section appears to have been handled splendidly. Although, it might be useful to discuss whether this threshold (≤40 for poor and ≥41 for good) was based on any empirical validation or theoretical consideration?

**Response:** The cut-off was theoretically determined. Please see data analysis section, line 192, page 9.

2. Were there any inter-coder reliability checks?

**Response:** We have added information about inter-coder reliability. Please see the data analysis section, lines 209-213, page 10.

Results

1. Participants’ characteristics: A brief discussion of the demographic diversity (e.g., age, sex, education level) and how representative it is of the broader population might be helpful.

**Response:** We opined that such details are unnecessary under results section to avoid repetitions of the table in words. However, we have discussed it under discussion section. Please see discussion section, lines 528-531, page 25.

2. Response distributions on PRHDS: More context around the interpretation of these scores would be helpful, such as what specific score ranges indicate in terms of risk perception.

**Response:** Information about the interpretation of the scores are provided under the instrument description and data analysis. We opined it is unnecessary here again to avoid repetition.

3. Sociodemographic predictors of CVD risk perception score: The adjusted R square value indicates that the model explains a modest portion of the variance in CVD risk perception, which is acceptable in social science research but should be noted as a limitation.

**Response:** We opined that this should not be noted as limitation. As you rightly noted, this is acceptable in social sciences. Moreso, we could not attribute any methodical flaw to the results.

4. Qualitative results: This is an outstanding and well-put-together section. However, a few points of note: The results are presented descriptively, but there is limited interpretation and discussion of the findings (more like limited in-text annotations to give proper context to the discussion). Finally, the format for presenting quotes is inconsistent. Some quotes are introduced with participant location, while others are not.

**Response:** We only introduced a quote based on location because some of the participants in both locations have divergent knowledge of CVD. However, we have improved the quotes. Please see the qualitative results.

Discussion

The section effectively synthesizes the quantitative and qualitative findings, showing how they complement each other. However, more emphasis could be placed on explaining the significance of these findings, particularly about public health interventions.

The comparison with other studies is thorough, and the differences in findings are well-explained. However, the discussion could delve deeper into why certain disparities exist. For example, the possible reasons behind the good risk perception in this study compared to others should be further explored. This could involve discussing cultural factors, differences in health literacy, or variations in public health messaging across regions.

The discussion on how urban dwellers have better CVD risk perception than rural dwellers is well-supported by qualitative and quantitative data. However, the potential reasons for these differences could be explored further. The role of access to healthcare, exposure to health information, and socio-economic factors could be discussed in more detail.

The discussion could benefit from integrating more theoretical perspectives on health behavior change and risk perception. For instance, how do the findings align with models such as the Health Belief Model or the Theory of Planned Behavior? This could provide a more robust framework for understanding the findings and their implications.

ii. Tone here could be more assertive: “This finding underscores the urgent need to enhance risk perception, particularly in rural areas."

iii. The discussion effectively highlights the importance of education in shaping CVD risk perception. However, the analysis could go further by discussing the role of other social determinants of health, such as income, employment status, and access to health services.

The gender differences in CVD risk perception are noted, but the discussion could benefit from a deeper exploration of why men in this study had better risk perception. Potential cultural, psychological, and social factors influencing this trend could be discussed more thoroughly.

iv. The clinical implications are relevant and well-argued, particularly the need for targeted public health interventions. However, the recommendations could be more specific. For example, what kind of public health campaigns would be most effective? How can healthcare providers be better equipped to improve CVD risk perception among rural populations?

**Response:** We have tried to improve the discussion incorporating the suggestions. Please see the discussion section, line 511-601, pages 24-28.

v. The discussion of limitations is concise but could be more comprehensive. For example, the impact of using a convenience sampling technique should be elaborated upon, especially in terms of how it might have skewed the results. Additionally, the limitations of self-reported data, such as recall bias, could be mentioned.

**Response:** We have expanded the limitation of the study. Please see the limitation section, lines 603-612, pages 28-29.

Conclusion

The conclusion effectively summarizes the key findings, particularly the differences in CVD perception between rural and urban dwellers. However, it could be more explicit in linking these findings to the broader context of public health in Nigeria. For example, how do these findings contribute to the understanding of CVD in low- and middle-income countries?

The conclusion rightly emphasizes the convergence between the quantitative and qualitative data, but it could also highlight any areas of divergence or unexpected findings, as this would provide a more nuanced understanding of the study results.

The conclusion could be further strengthened by including a forward-looking statement about future research directions. For example, what gaps in knowledge remain, and how should future studies address them? This would provide a more comprehensive end to the study and suggest pathways for continued inquiry.

**Response:** These have been incorporated. Please see the conclusion and recommendation sections, lines 620-630, pages 29=30.
